# Geochemical characterization of Lucaogou Formation and its correlation of tight oil accumulation in Jimsar Sag of Junggar Basin, Northwestern China

**DOI:** 10.1007/s13202-017-0335-1

**Published:** 2017-03-09

**Authors:** Jiangxiu Qu, Xiujian Ding, Ming Zha, Hong Chen, Changhai Gao, Zimeng Wang

**Affiliations:** 1 School of Geosciences in China University of Petroleum, Qingdao, 266580 China; 2 Laboratory for Marine Mineral Resources, Qingdao National Laboratory for Marine Science and Technology, Qingdao, 266580 China; 3 Research Institute of Petroleum Exploration and Development, Xinjiang Petroleum Administration Bureau, Karamay, 841000 China; 4 No. 66, Changjiang West Road, Huangdao District, Qingdao, 266580 China

**Keywords:** Tight oil, Source rock, Lucaogou Formation, Jimsar Sag, Junggar Basin

## Abstract

With the constant consumption of conventional oil and gas resources, unconventional oil and gas resources with great resource potential such as tight oil have gradually been valued and become the new exploration area. Jimsar Sag is the key tight oil exploration and development block in Junggar Basin of Northwestern China. Based on the data sets of geology, oil production test, logging, rock thin section, and geochemistry of Permian Lucaogou Formation (LCG), we studied the geochemical characteristics of hydrocarbon source rocks and their relation to the tight oil accumulation. Organic matter abundance of source rocks is high, the types of organic matter are mainly type I and type II, and the organic matter maturation is in the low mature stage to mature stage. Results of oil source correlation showed that the crude oil of sweet spots was mainly derived from the source rocks in the interior of the sweet spots. The LCG tight oil is mainly distributed in the plane where the source rocks have great thickness and the TOC is higher than 3.5%. It shows that the source rocks have obvious controlling on the occurrence and accumulation of tight oil.

## Introduction

With the successful exploitation of Bakken oil (Williston Basin, North America; Miller et al. 2008) and Eagle Ford oil (South Texas) (Mullen 2010), tight oil has been a research focus in global petroleum geology (Johnstone 2007; Zou et al. 2013; Hill et al. 2007) and was considered the most practical unconventional replacement for oil and gas besides shale gas (USGS 2008; Cao et al. 2016a, b). China is abundant in tight oil resources, as a new round of national petroleum resources assessment jointly completed by Land and Resources and other ministries thinks that tight oil favorable exploration area in China is (41 ~ 54) × 10^4^ km^2^, and the amount of geological resources is up to 200 × 10^8^ t, accounting for 2/5 of the recoverable oil resources (Wang 2013; Zou et al. 2014; National Key Basic Research and Development Program (973 Program) 2014); thus, tight oil has become one of the most realistic unconventional oil exploration areas (Wang 2013; Jia et al. 2012; Cao et al. 2016a).

In recent years much progress has been made in tight oil exploration of the Permian Lucaogou (LCG) Formation, which is the primary exploratory stratum of tight oil reservoirs in the Jimsar Sag, Junggar Basin, Northwestern China. Jimsar Sag is located at the eastern uplift of Junggar Basin and covers an area of 1278 km^2^. The Lucaogou Formation in Jimsar Sag, which formed in the sedimentary environment of a lacustrine basin, is a set of mixed fine-grained deposits. Mudstones, siltstones, sandstones, and dolomites are the main rock types with the characteristics of fine grains, thin single layers, and frequently alternating lithology (Si et al. 2013; Li et al. 2014). Although the LCG Formation generally has low porosity and low permeability, the physical properties and oil-bearing probability in some intervals are relatively better. Tight oil is widely distributed in Jimsar Sag, in which commercial oil with high production has been obtained in multiple wells and has a great prospecting potential (Kuang et al. 2012, 2013a, b).

Currently, the study on geochemical characterization of LCG Formation and its correlation of tight oil accumulation in Jimsar Sag of Junggar Basin is still relatively weak. We have conducted a comprehensive investigation on the source rock of LCG Formation, to define the geochemical characterization of LCG Formation and its correlation of tight oil accumulation in the Jimsar Sag of Junggar Basin.

## Geological setting

**Fig. 1:**
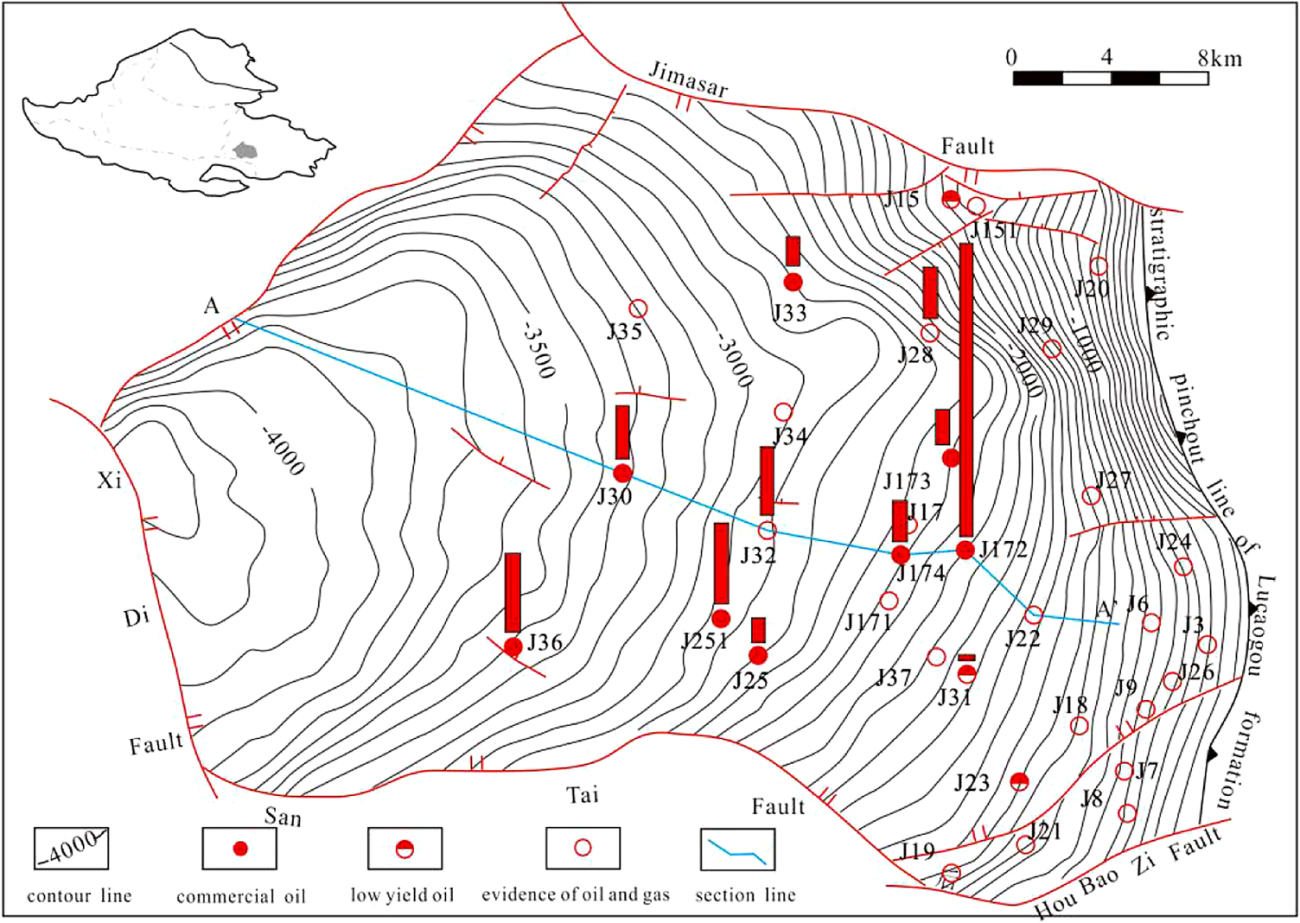
Map of tight oil exploration results of LCG Formation in the Jimsar Sag, Junggar Basin

The Jimsar Sag is located in the eastern uplift of Junggar Basin, which is one of the most petroliferous basins with great proven oil reserves and undiscovered resources in the Northwestern China (Fig. 1). The Sag has a total area of 1278 km^2^ and is a faulted depression with faulting in the west, and it overlies the Middle Carboniferous folded basement in the east. The tectonic activity in the inside of the depression is relatively weak (Zhao et al. 1994; Zou et al. 2014), and body exploration site is gentle with 3°–5° structural dip. Recently, commercial tight oil flow has been obtained from many oil wells, and the sag has been the focus of tight oil exploration and development in China (Wang et al. 2015; Jiang et al. 2015; Cao et al. 2016b, 2017).

**Fig. 2:**
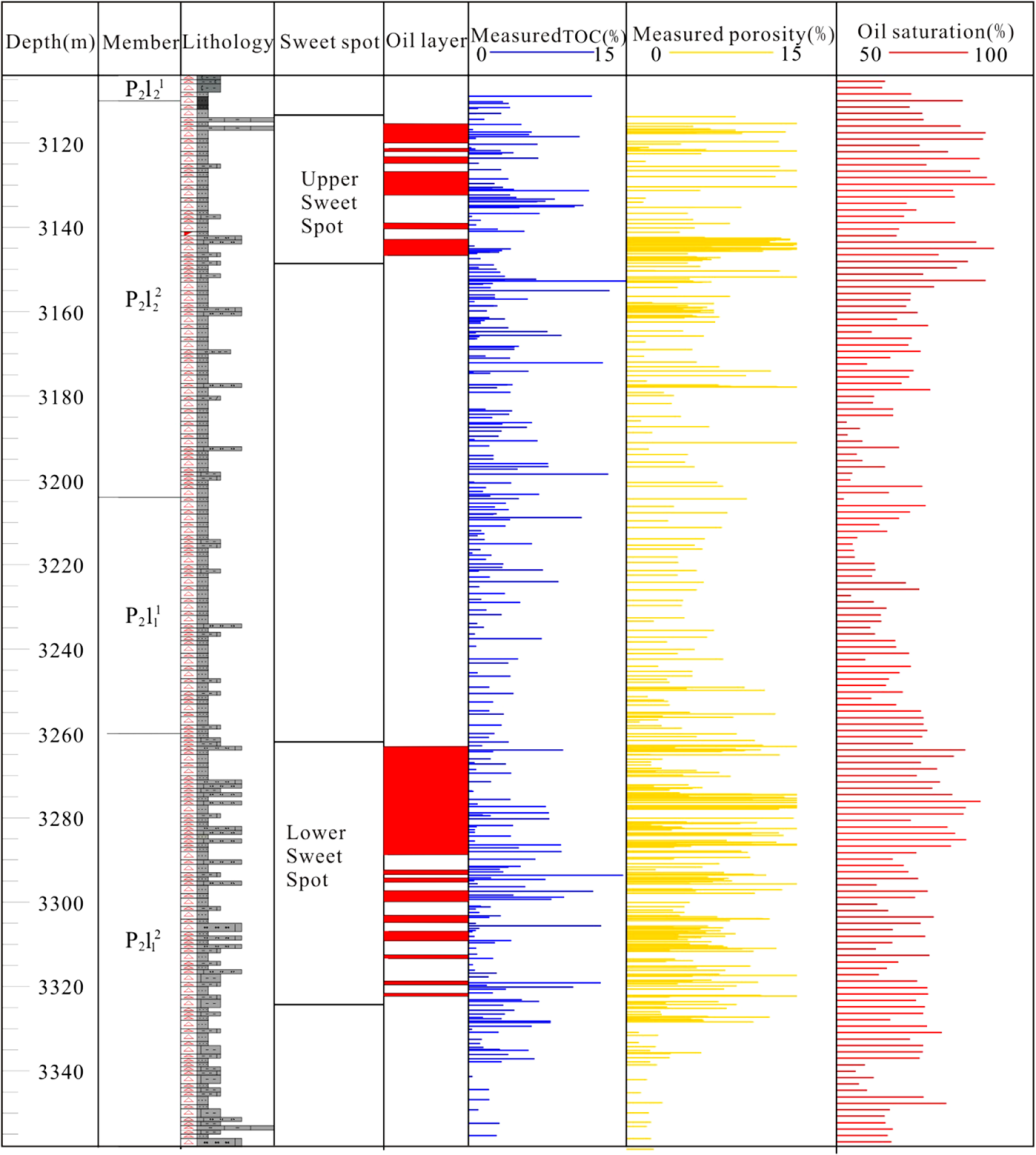
Generalized stratigraphic column of LCG Formation in the Jimsar Sag, Junggar Basin

The sag includes sedimentary cover from Permian to Quaternary with Middle Carboniferous flexure as the basement. LCG is the main target layer for tight oil exploration, which is mainly deposited in lacustrine environment (Kuang et al. 2013a, b; Zhang et al. 2003; Peng et al. 2011). LCG can be further subdivided into the lower Lucaogou Member (P_2_l_1_) and the upper Lucaogou Member (P_2_l_2_) (Jiang et al. 2015). Tight oil is mainly distributed in the sweet spots of P_2_l_1_ and P_2_l_2_, which is shown in Fig. 2.

## Materials and methods

The source rock samples in the Lucaogou Formation were analyzed in this study. They were collected from Jimsar Sag in the Junggar Basin. One sample set consists of 300 mudstone samples from different sedimentary facies. This sample set is pulverized to 80 meshes in preparation for Rock-Eval pyrolysis, total organic carbon content (TOC). A Rock-Eval instrument is used to perform the pyrolysis analysis, which provides the parameters of *T*_max_, *S*_1_, and *S*_2_. The temperature was set as 300 °C for 3 min and then heating at 25 °C/min to 650 °C (Ding et al. 2015). *T*_max_ is the temperature which corresponds to the maximum generation rate of hydrocarbons from kerogen cracking. *S*_1_ and *S*_2_ represent the amount of hydrocarbon of free and generated hydrocarbons, respectively. The TOC of the source rocks is measured using a LECO CS-230 analyzer.

Twenty-nine mudstones are used for gas chromatography–mass spectrometry (GC–MS) analysis. In order to assess organic matter type and depositional condition more accurately, the limited mudstone samples are selected as dispersedly as possible from Jimsar Sag. The rock samples are cleaned prior to powdering. Soxhlet extraction was conducted using chloroform/methanol (87:13) for 72 h, and the isolated extractable organic matter was separated into saturated hydrocarbons, aromatic hydrocarbons, and polars using column chromatography method. GC–mass spectrometry (GC–MS) analysis of the saturate fractions is performed with a HP6890GC/5973MSD instrument equipped with a HP-5MS fused silica column (30 m × 0.25 mm i.d., film thickness 0.25 mm). The G-Coven temperature for analysis of the saturate fractions is initially held at 50 °C for 2 min, then programmed to 100 °C at 20 °C/min and to 310 °C at 3 °C/min, and held at 310 °C for 16.5 min. Biomarker ratios are calculated using peak areas of individual compounds. Carbon isotopic compositions of carbonate in the mudstone samples are determined by a traditional acid-release method. Powdered samples are treated with anhydrous H_3_PO_4_ at 25 °C for 24 h to liberate CO_2_, and the liberated CO_2_ is collected and sealed for carbon isotope analysis. The carbon isotopic ratio is analyzed on a Finnigan MAT 252 mass spectrometer. Results are reported in standard per mil d-notation relative to the V-PDB standard. The error of these analyses is less than 0.1‰. The results are listed in Table 1.

## Results

### Organic matter abundance and Rock-Eval pyrolysis

LCG source rocks exhibit a wide range in TOC contents, laterally from 0.53 to 12.42%, and most of the TOC contents are less than 5% (Table 1).

**Table 1:** Geochemical parameter table of LCG Formation in the Jimsar Sag, Junggar Basin

Depth (m)	*T*_max_	TOC	*S*_1_	*S*_2_	20S (%)	ββ (%)	%C_27_	%C_28_	%C_29_	Pr/Ph
3110.88	448	3.55	0.66	8.35	0.982	0.009	0.151	0.334	0.515	1.860
3112.09	443	0.85	0.42	1.92	0.996	0.003	0.223	0.310	0.467	1.050
3113.3	441	2.89	1.96	10.11	0.978	0.011	0.188	0.327	0.485	1.350
3113.34	443	0.72	0.02	0.71	0.998	0.002	0.181	0.341	0.478	1.630
3114.73	451	1.42	0.28	3.87	0.991	0.004	0.328	0.296	0.376	1.030
3117.1	439	0.39	0.01	0.14	1.000	0.001	0.167	0.339	0.494	1.230
3117.75	449	5.57	0.53	22.48	0.952	0.013	0.216	0.336	0.448	2.260
3118.78	453	9.77	0.44	78.96	0.852	0.019	0.187	0.342	0.472	1.240
3119.23	442	0.65	0.02	1.39	0.997	0.002	0.167	0.334	0.499	2.110
3121.38	444	0.27	0.03	0.5	0.999	0.001	0.148	0.346	0.506	0.970
3122.14	448	2.84	0.49	8.85	0.981	0.007	0.146	0.315	0.539	1.440
3122.58	450	3.96	0.33	17.32	0.963	0.009	0.118	0.402	0.480	1.750
3125.08	443	0.92	0.01	1.32	0.997	0.002	0.152	0.352	0.496	1.220
3130.76	445	3.03	0.4	4.16	0.991	0.008	0.271	0.311	0.418	1.330
3134.05	444	6.77	0.74	32.75	0.931	0.016	0.230	0.330	0.440	1.260
3134.21	448	6.05	0.88	22.19	0.953	0.015	0.224	0.314	0.462	1.100
3137.01	452	6.25	0.36	32.9	0.932	0.013	0.212	0.367	0.421	1.210
3139.7	444	0.72	0.03	2.12	0.995	0.002	0.145	0.353	0.502	0.970
3140.74	450	2.58	0.34	6.89	0.985	0.006	0.138	0.364	0.498	1.110
3144.66	442	0.55	0.06	0.99	0.998	0.001	0.143	0.360	0.496	1.030
3150.2	448	2.48	0.64	3.2	0.993	0.007	0.235	0.407	0.357	0.840
3152.82	451	3.17	0.02	20.2	0.957	0.007	0.139	0.587	0.274	1.600
3153.65	453	2.14	0.12	1.2	0.997	0.005	0.173	0.477	0.349	2.970
3155.32	452	12.42	0.65	176	0.720	0.020	0.138	0.607	0.255	0.970
3156.94	453	2.32	0.15	4.32	0.991	0.005	0.124	0.382	0.494	1.440
3158.88	444	2.54	0.24	12.95	0.972	0.006	0.194	0.417	0.389	0.910
3161.75	448	1.85	0.02	10.85	0.976	0.004	0.201	0.450	0.349	0.740
3162.02	453	3.22	0.17	10.76	0.977	0.007	0.184	0.438	0.378	0.810
3162.62	448	1.08	0.15	0.81	0.998	0.003	0.207	0.294	0.499	0.930

*S*_*1*_ *(mg/g)* the amount of hydrocarbon of free, *S*_*2*_ *(mg/g)* the amount of generated hydrocarbons; %27 = C_27_ steranes/(C_27_ steranes + C_28_ steranes + C_29_ steranes); %28 = C_28_ steranes/(C_27_ steranes + C_28_ steranes + C_29_ steranes); %29 = C_29_ steranes/(C_27_ steranes + C_28_ steranes + C_29_ steranes); 20S(%) = C_29_ sterane ααα20S/(20S + 20R); ββ(%) = C_29_ sterane αββ/(αββ + ααα)

**Fig. 3:**
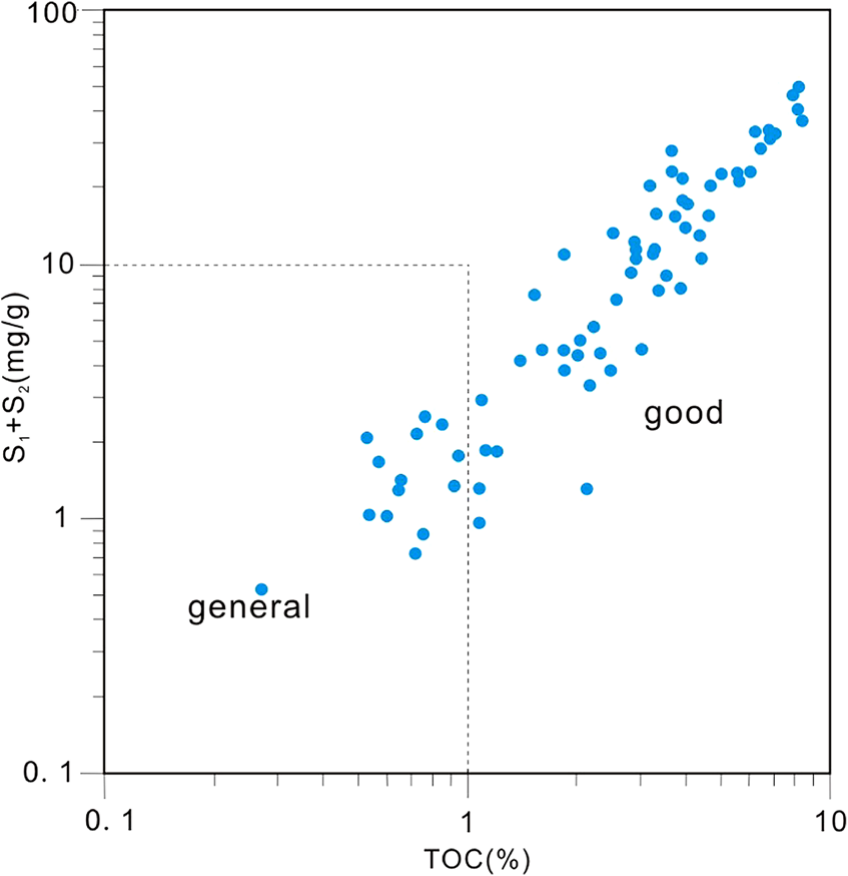
Cross-plot of the total organic matter content (TOC) versus *S*_1_ + *S*_2_, showing the high organic matter ambulance of LCG source rock in the Jimsar Sag of Junggar Basin

Rock-Eval pyrolysis is a commonly used technique to assess hydrocarbon generating potentials and classify organic matter types (Peters 1986). The average value *S*_1_ + *S*_2_ of source rock samples is 12.93 mg/g, in the range of 4.96 ~ 176.65, showing that the source rocks have high organic matter abundance (Table 1). The LCG Formation is high in organic matter abundance, and it belongs to good source rock with fairly high hydrocarbon generation conditions (Fig. 3).

#### Organic matter types

**Fig. 4:**
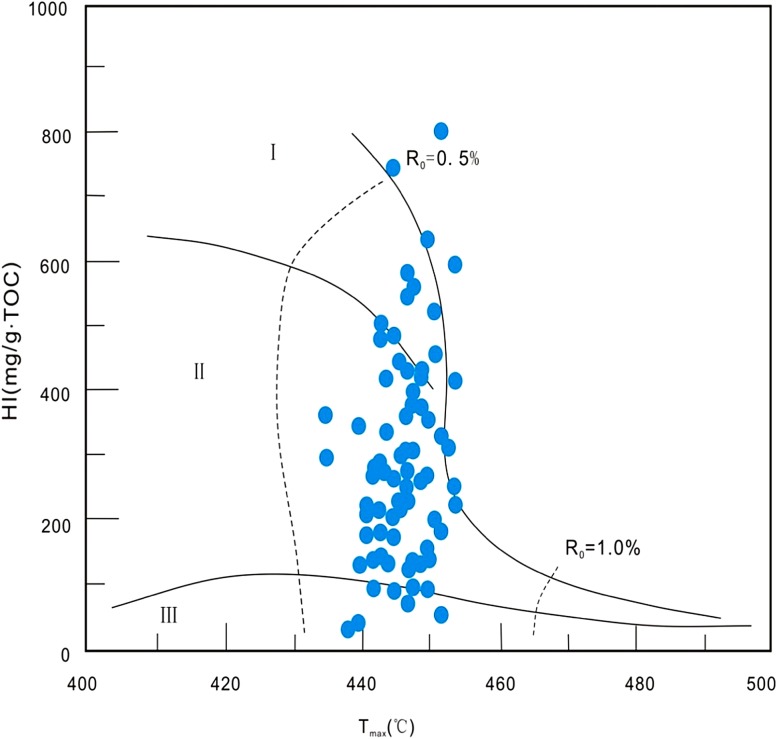
Cross-plot of *T*_max_ versus HI (*S*_2_/TOC), showing the organic matter type of LCG source rock in the Jimsar Sag of Junggar Basin

The hydrocarbon potential of source rocks is not just about the hydrocarbon source rock organic matter abundance, but also the type of organic matter in the source rocks. Cross-plots of *T*_max_ versus HI (*S*_2_/TOC) show that LCG source rocks are dominated by type I and II kerogen (Fig. 4).

#### Organic matter maturity

**Fig. 5:**
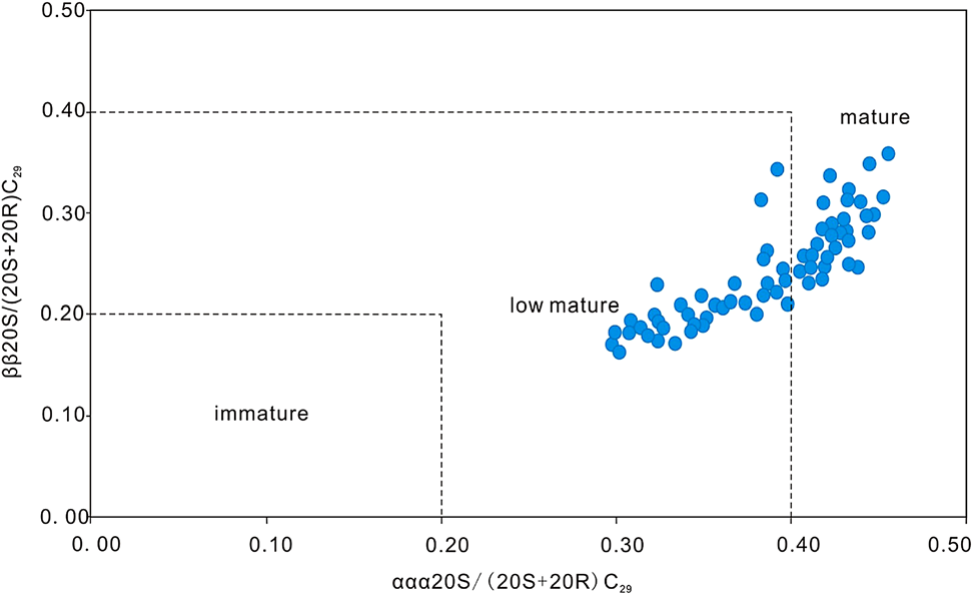
Characteristics of the maturity parameter of soluble organic matter C_29_ regular sterane of Lucaogou Formation in Jimsar Sag

Most of source rock samples have Rock-Eval *T*_max_ of 435–455 °C, suggesting a low maturity to maturity stage (Table 1). The biomarker maturity parameters ααα20S/(20S + 20R) C_29_ sterane and ββ/(αα + ββ) C_29_ sterane parameters indicate that LCG source rocks are mostly in the low maturity to mature evolution stage (Fig. 5).

### Oil source correlation

**Fig. 6:**
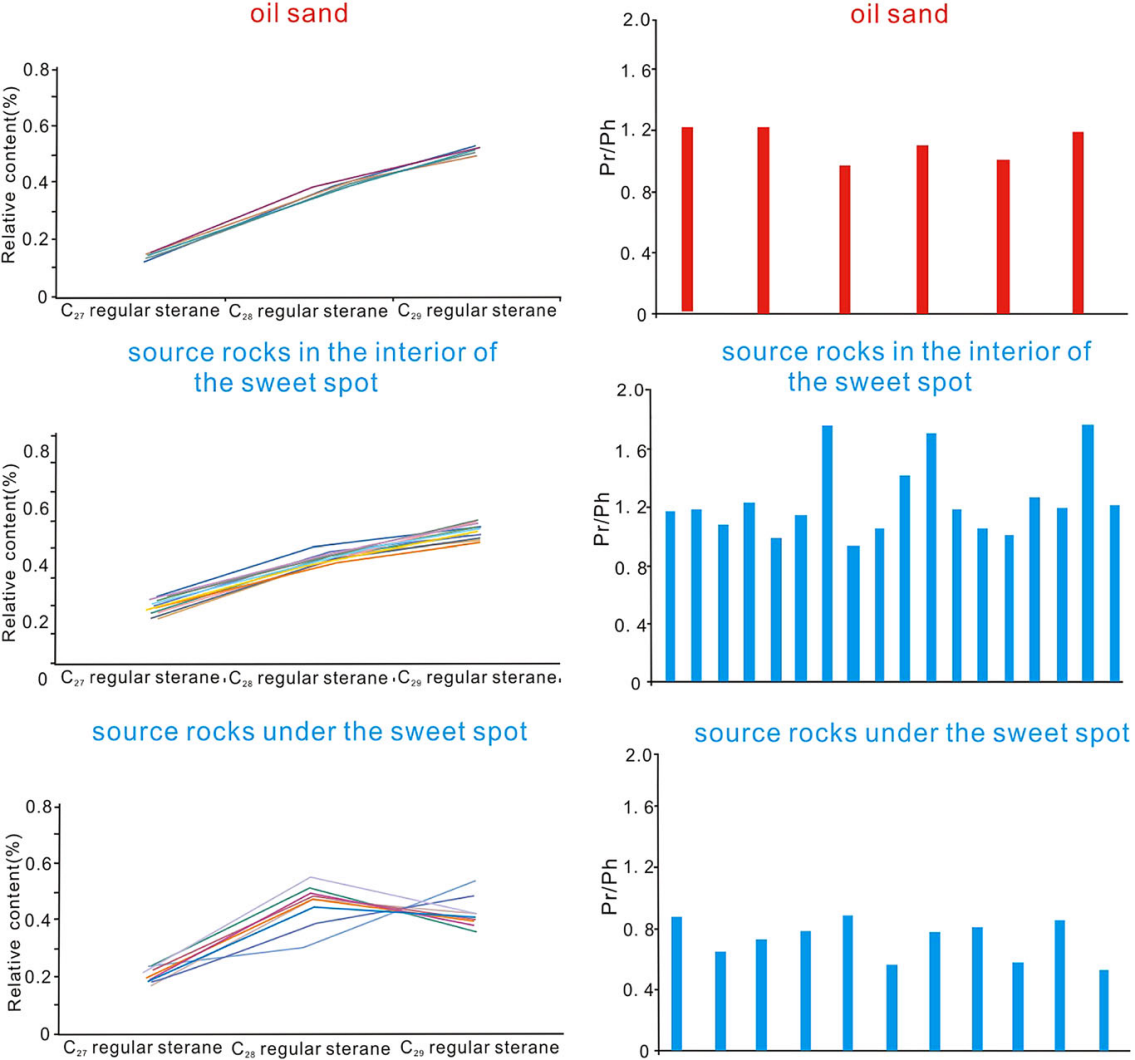
Crude oil and the source rock geochemical characteristics of *upper* sweet spot in LCG Formation

Biomarkers have drawn much attention in the past few decades, mainly because of their usefulness in assessing organic matter type and quality, depositional conditions (e.g., salinity, oxicity, anoxicity), thermal maturity level, biodegradation extent and lithology (Arfaoui et al. 2007; Ding et al. 2016). In the upper sweet spot, the source rocks in the interior of the sweet spot are characterized by high value of Pr/Ph (>1), low C_27_ steranes, moderate C_28_ steranes, and high C_29_ steranes, which are consistent with the biomarkers characteristics of oil in the upper sweet spot (Fig. 6). And the source rocks under the upper sweet spot display low value of Pr/Ph (<1), low C_27_ steranes, high C_28_ steranes, and moderate C_29_ steranes (Fig. 6).

**Fig. 7:**
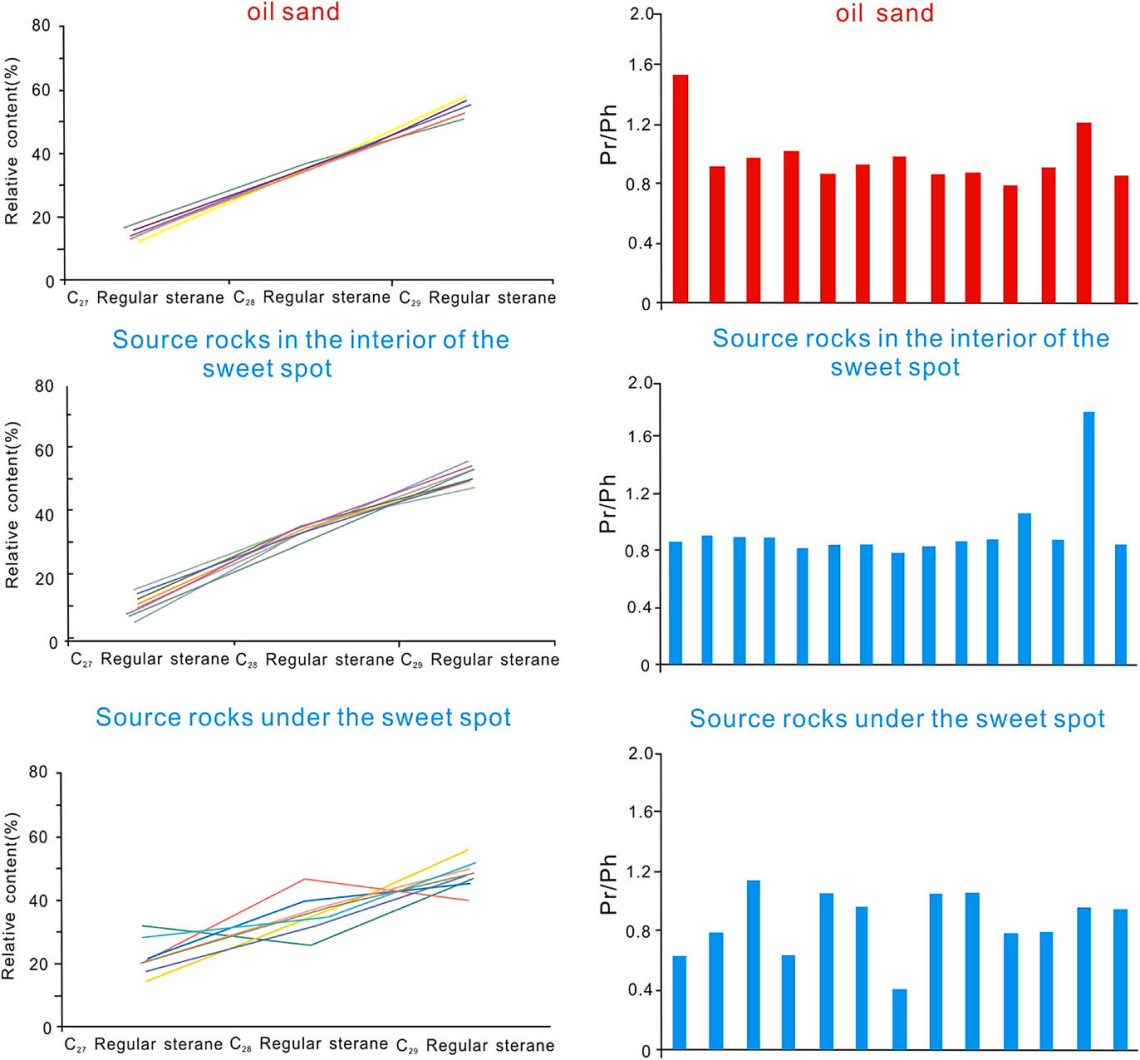
Crude oil and the source rock geochemical characteristics of *lower* sweet spot in LCG Formation

In the lower sweet spot, the source rocks in the interior of the sweet spot are characterized by high value of Pr/Ph (>0.8), low C_27_ steranes, moderate C_28_ steranes, and high C_29_ steranes, which is consistent with the biomarker characteristics of oil in the lower sweet spot. And the source rocks under the lower sweet spot display high value of Pr/Ph (> 0.8), low C_27_ steranes, high C_28_ steranes, and moderate C_29_ steranes. The analysis of the biomarker distribution indicates that the tight oil migration is dominated by primary or short-distance migration (Fig. 7).

## Discussion

LCG tight oil is mainly distributed in the upper and lower sweet spot, where in upper sweet spot source rocks TOC is mainly distributed in the range of 2–8%, lower sweet spot source rocks TOC is mainly distributed in the range of 0.5–6%, and non-sweet spot hydrocarbon source rocks TOC is mainly distributed in the 0.5–4%. Hydrocarbon source rocks abundance is significantly lower than the sweet spot, indicating that Lucaogou Formation tight oil is mainly distributed in the high organic abundance of source rocks symbiotic tight reservoirs, and high quality mature hydrocarbon source rocks inside or adjacent to tight reservoir is mainly tight oil distributed layer system.

**Fig. 8:**
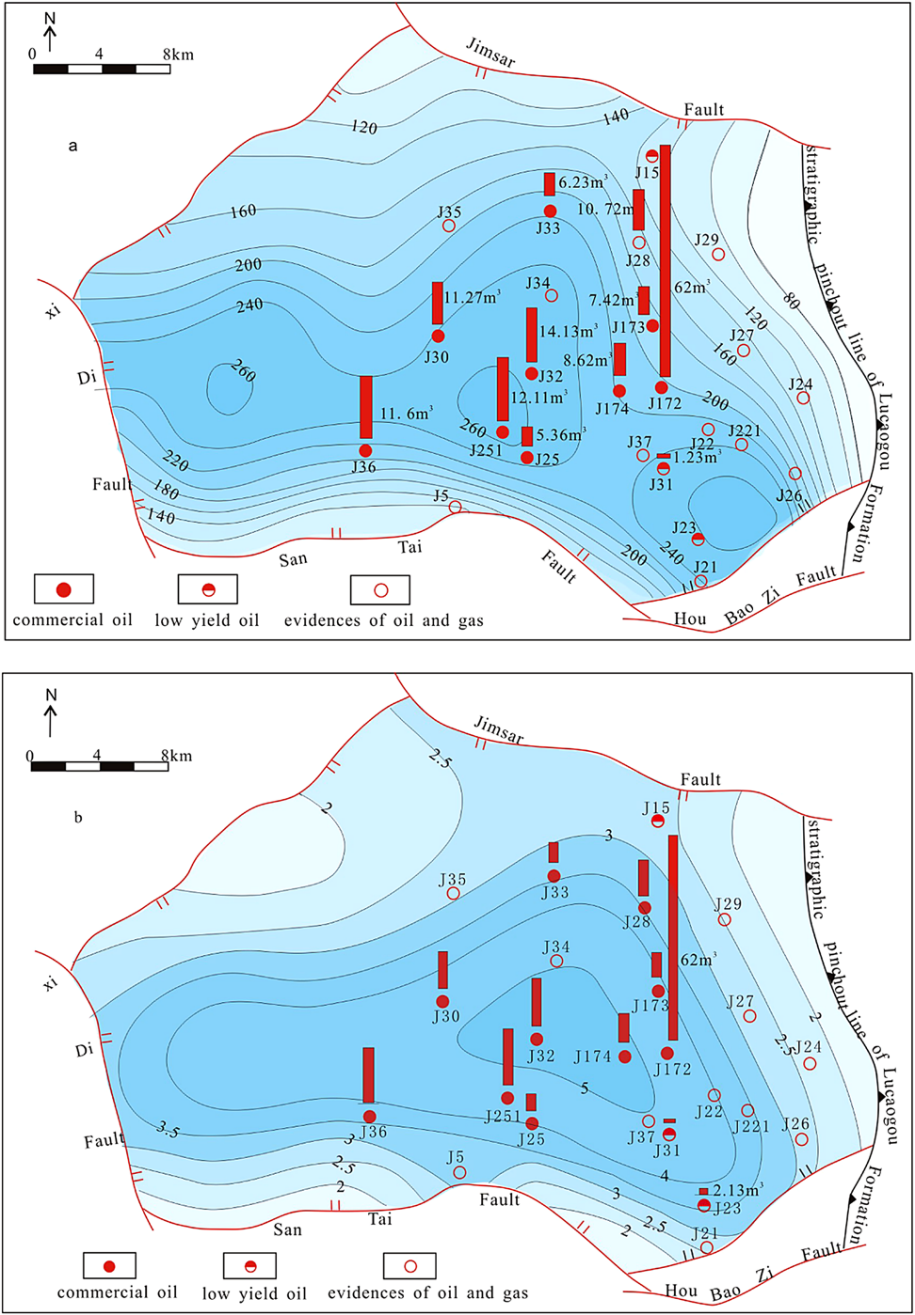
Source rocks and tight oil distribution of LCG Formation in the Jimsar Sag, Junggar Basin: **a** source rocks thickness; **b** TOC of source rocks

The LCG tight oil accumulation is controlled by source rock and proved by that tight oil is mainly distributed in the region with thick source rocks and high abundance of organic matter. LCG Formation tight oil is mainly distributed in the region with hydrocarbon source rock accumulated thickness of more than 200 m region, where many commercial oil flow wells are located, such as J 174 well and J 251 well (Fig. 8a). And there are many evidence oil and gas wells in the region with source rock thickness of less than 200 m, such as J 27 well, J 29 well and J 35 well. The organic matter abundance also has significant control on tight oil distribution which is mainly distributed in the area of source rocks with TOC higher than 3.5% (Fig. 8b).

## Conclusion

1. The average total organic carbon content and hydrocarbon generating potential of LCG source rocks are 3.36% and 15.61 mg/g, respectively, which have reached the standards for “good” hydrocarbon source rock. The LCG organic matter is dominated by type I and II kerogen and in the low maturity to mature evolution stage.
2. The analysis of the biomarker distribution indicates that the tight oil migration is dominated by primary or short-distance migration. In the upper sweet section, the tight oil and source rock both display high value of Pr/Ph (>1), low C_27_ steranes, moderate C_28_ steranes, and high C_29_ steranes. And in the lower sweet spot, the tight oil and source rock also show the same characteristics.
3. It is concluded that the LCG tight oil accumulation is controlled by source rocks, which is proved by that oil is mainly distributed in the region with thick source rocks and high abundance of organic matter.
